# Investigation into the Effects of Crystalline Admixtures and Coatings on the Properties of Self-Healing Concrete

**DOI:** 10.3390/ma17030767

**Published:** 2024-02-05

**Authors:** Ravi Kumar Shetiya, Sara Elhadad, Ali Salem, Attila Fülöp, Zoltan Orban

**Affiliations:** 1 Structural Diagnostics and Analysis Research Group, Faculty of Engineering and Information Technology, University of Pécs, 7622 Pécs, Hungary; ravishetiya1999@gmail.com (R.K.S.); salem.ali@mik.pte.hu (A.S.); fulop.attila@mik.pte.hu (A.F.); orban.zoltan@mik.pte.hu (Z.O.); 2 Department of Architecture, Faculty of Engineering, Minia University, Minia 61111, Egypt; 3 Civil Engineering Department, Faculty of Engineering, Minia University, Minia 61111, Egypt

**Keywords:** self-healing concrete, fresh properties, hardened properties, crystalline admixture, durability

## Abstract

One fascinating concept for enhancing the durability and lifespan of concrete buildings involves the use of self-healing concrete. This study focuses on the effect of crystalline admixtures and coatings on various properties of self-healing concrete and provides a comparison with traditional concrete. Four different concrete mixtures were prepared to assess their effectiveness in bridging crack openings, their flexural and compressive strengths, and water absorption. Various testing methods, including destructive, semi-destructive, and non-destructive tests, were used in this research. The capacity of the mixes to repair themselves was assessed on the destroyed and semi-destroyed test specimens using crack-healing and microstructure testing. Additionally, all mixtures were also subjected to the slump cone test and air content test in order to investigate the characteristics of the concrete in its fresh state. The findings demonstrate that crystalline coating and admixture combinations have significant potential for healing concrete. The compressive and bending strengths of self-healing concrete mixtures were shown to be slightly higher compared to traditional concrete when the additive dose was increased. Self-healing concrete mixtures also exhibited much lower water absorption, a tightly packed and improved microstructure, and signs of healed gaps, all of which indicate greater durability.

## 1. Introduction

Concrete is widely utilized in the construction industry owing to its readily accessible nature, economical price, and favorable mechanical properties. The advent of reinforced concrete has brought about substantial changes in the course of architectural and engineering progress due to its inherent durability and the integration of steel reinforcement.

Nevertheless, emerging environmental problems and climate change, have raised significant issues about the manufacturing and utilization of concrete. The large-scale manufacturing and application of concrete results in significant carbon dioxide emissions, water pollution, waste generation, and the depletion of natural resources. When cement factories calcinate raw materials, such as clay and limestone, they emit large amounts of carbon dioxide into the atmosphere. Other associated processes, such as burning and grinding clinker, and the transportation of raw materials and finished products, also contribute to climate change.

The generation and proper disposal of concrete debris following the demolition of a structure also pose significant ecological challenges. Construction and demolition activities account for a significant proportion of the EU’s waste, which includes a wide range of materials such as concrete, plastic, wood, glass, and metals, etc. The amount of this waste is believed to be between twenty-five and thirty percent of the total waste disposed of in Europe. While the recycling of construction and demolition waste may appear feasible in theory, its widespread implementation is hindered by practical challenges.

The importance of implementing environmentally friendly policies in the construction industry is underscored by the variety of challenges posed by the increased carbon dioxide emissions that lead to climate change, water pollution, and waste generation. Nevertheless, the environmental repercussions of concrete go beyond its production and disposal. Defects, such as cracks and fractures in the structure of a building, can have serious social and economic consequences, in addition to environmental impacts. In addition to compromising concrete’s structural integrity, cracking increases the demand for either the construction of new structures or restoration, both of which have significant environmental, as well as social and financial, costs.

Cement-based mixtures typically exhibit shrinkage cracks [1,2]. Concrete is a mixture of materials: a cement matrix plus coarse and fine aggregates. Theoretically and empirically, it has been established that the shrinking of concrete results in the emergence of a network of small and large fractures surrounding both fibers, as well as aggregates [1,2]. The major causes of the fractures in concrete are a relatively weak tensile strength, temperature and shrinkage distortions, ingression of harmful chemicals, freeze–thaw cycles, foundation imperfections, and settlement [3,4].

Since cracks permit detrimental fluids to flow through the fissures, they decrease the building material’s durability over time [5]. Tiny cracks in concrete can initiate deterioration, and liquid seeping through the building material may further affect reinforcement steel [6]. Hence, it is vital that these fractures are quickly repaired. The ability of concrete structures to heal from fractures increases their long-term viability and durability and makes them more sustainable [7].

Concrete’s inherent self-healing abilities have made it a renowned and a widely recognized construction material [5,6,7,8]. Attempts to develop autonomous self-healing concrete have been ongoing since the 1990s [6,7,8]. Henk Jonkers, a microbiology researcher from Delft University of Technology in the Netherlands, developed self-healing concrete, an entirely innovative form of concrete, in around 2006 [8,9]. Self-healing concrete encompasses a unique mechanism which is frequently referred to as the concrete’s capacity to repair itself or to autonomously heal after cracking. Another name for this material is self-repairing concrete [10,11,12,13,14,15]. Self-repairing mimics the human’s inherent capacity to restore itself by secreting a specific type of fluid [7,8,9,10,11].

Recently, a number of self-healing techniques have been introduced [4,5,6,7,8], such as the incorporation of crystalline additives [12,13,14,15,16], microencapsulating repair agents [17,18,19,20,21,22,23], and bacteria [24,25,26,27,28,29,30]. When cracks develop, these substances become active and react or release fluids to fill the gaps [8]. Self-repairing in cementitious materials, based on methods derived from self-repairing polymers, can be divided into three groups: intrinsic healing, capsule-based healing, and vascular healing [7,31]. Autogenous repair is recognized as one of the most extensively researched methods for the intrinsic repair of cracks in cement-based products [32,33,34,35,36,37]. Two processes are primarily responsible for autogenous fracture healing [7,8]: (1) hydration of unhydrated cement molecules, and (2) the dissolution and associated carbonation of $\mathrm{Ca}{\left(\mathrm{OH}\right)}_{2}$ [7,9,38]. Young concrete is known to have the highest self-repair capability [4].

Different metrics can be employed to evaluate the effectiveness of self-repair mechanisms: visible crack closure assessment, identification of the responsible repair ingredients, enhanced durability, as well as the restoration of strength capabilities [39,40]. However, concrete’s ability to rebuild its strength throughout the self-repair phase is often minimized [41,42]. As a result, self-treatment activity tends to be apparent when visible crack closure is observed and durability improvements are achieved, as demonstrated by durability tests and microstructural analyses [43].

There is an increased interest in self-repairing materials, particularly those with self-healing qualities in ecologically friendly and sustainable construction materials, with a focus on diverse approaches offered by several research works over the past 20 years. However, it is difficult to choose the most effective testing technique since every experiment uses a different set of testing procedures to gauge how well the repairs work. Concrete that can mend itself has the capacity to regenerate, which lessens the need to locate and correct internal flaws (like gaps) without outside intervention. Consequently, cost decreases, durability increases, and corrosion of the concrete, and reinforcement, are also limited.

In addressing the adverse environmental impacts and associated challenges inherent in the use of conventional concrete, this study focuses on the potential of self-healing concrete. The primary objective is to examine the effectiveness of crystalline admixtures in enhancing the self-healing properties of concrete, with the further goal of mitigating the environmental impacts of construction materials and supporting initiatives to promote sustainability in the industry.

The goal of this research is to conduct a series of experiments on four different mixes, involving three varieties of self-healing concrete and typical ordinary concrete, in order to evaluate how efficiently they perform in terms of a number of fresh and hardened properties, including slump, air content, rebound value, water absorption, compressive strength, flexural strength, etc. The crack width, crack depth and the microstructure of the hardened concrete specimens are also analyzed. By comparing the outcomes of these distinct mixes and the ingredients that compose them, this study aims to shed light on the effectiveness of crystalline admixtures (CA) and coatings in self-healing concrete and provide guidance regarding the future advancement and implementation of this innovation in the building industry.

## 2. Materials and Methods

### 2.1. General

**Figure 1:**
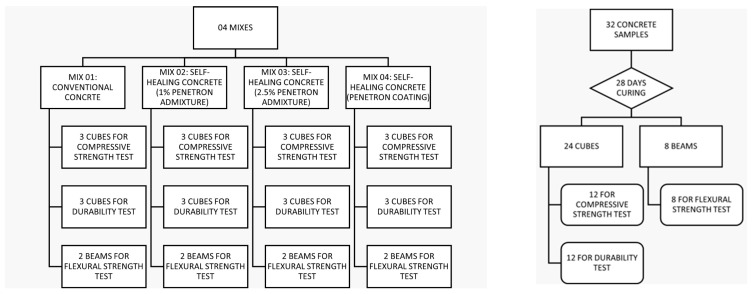
Research methodology.

**Figure 2:**
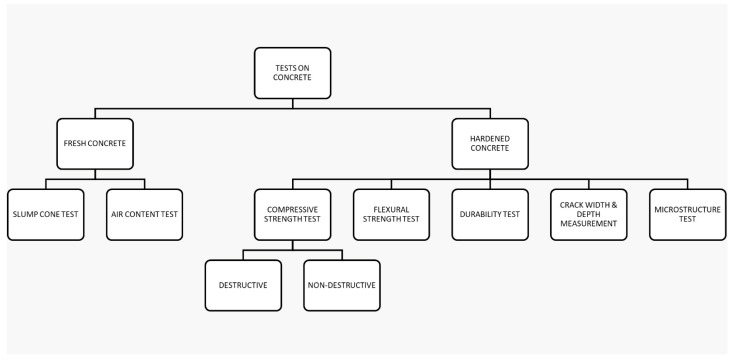
Tests on concrete.

The goal of this work is to determine how crystalline admixtures and coatings affect the capacity for self-healing in concrete. For the study, four separate mixtures were formulated, including a standard mixture and three self-healing mixtures, manufactured with differing concentrations of Penetron crystalline admixture and coating (Figure 1). In the fourth mix, the crystalline coating is applied over concrete specimens made using the conventional mix. A range of destructive, non-destructive, and semi-destructive testing methods were used to evaluate the performance of the mixes, including compressive strength, flexural strength, durability, and microstructure investigations (Figure 2). The features associated with fresh concrete, including slump and air content, were also investigated. Measurements of the depth and width of cracks were used to gauge the self-healing ability of the different combinations.

### 2.2. Mix Design

Standard OPC CEM I 42.5 N was used as the binder, along with potable water in the mix. For Mix 4, the crystalline coating was prepared by adding water to the crystalline admixture (water to admixture ratio = 0.4) that was applied on the damp surface of concrete in two coats, 6 h apart and 28 days after curing. The four mixtures are presented in Table 1:

Six 15 cm cubes were cast for each mixture; three of these cubes were used for testing compressive strength and three for testing durability. Additionally, two prismatic beams measuring 15 cm × 15 cm × 60 cm were cast and tested for flexural strength. Each sample underwent a curing process for 28 days before testing.

### 2.3. Tests on Concrete

#### 2.3.1. Tests on Fresh Concrete

**Figure 3:**
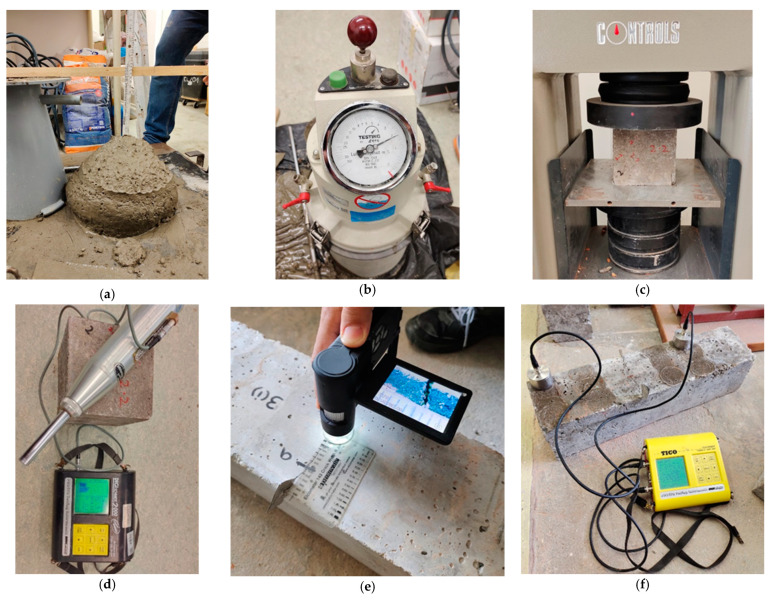
Tests on concrete: (**a**) slump cone test; (**b**) air content test; (**c**) destructive compressive strength test; (**d**) non-destructive compressive strength test; (**e**) crack width measurement; and (**f**) crack depth measurement.

The slump test and the air content test were carried out in accordance with standard testing practices (see Figure 3a,b) in order to assess the workability and air content of the fresh concrete.

#### 2.3.2. Tests on Hardened Concrete

- Compressive Strength Tests

Both destructive and non-destructive tests were employed to evaluate the specimens’ compressive strengths. First, each sample was subjected to the non-destructive rebound hammer test (Refer Figure 3d) to determine its compressive strength. A typical destructive test was then conducted. For destructive compressive strength testing, the cubes were loaded at a rate of 3000 Ns, gradually and constantly until they failed, as shown in Figure 3c;

- Flexural Strength Test

Flexural testing on beam samples with dimensions of 15 cm by 15 cm by 60 cm was conducted using a 4-point bending system. The supporting span (Lo) was maintained at 45 cm, while the loading span was maintained at 15 cm. A loading rate of 150 Ns was applied until the first crack appeared, with a 1 mm opening (visually regulated). Following the preliminary bending test, Mix 4 specimens were coated with the crystalline admixture and all specimens were kept in water for 12 days. The modulus of rupture was estimated using the following expression:

(1) $$MR=\frac{PL}{bd^{2}};$$

- Water Absorption Test

As the durability of concrete structures depends largely on their ability to absorb water, a water absorption test was conducted to analyze this durability-related characteristic of all mixes. The test cube samples were placed in the oven and heated to a controlled temperature of 120 °C for 24 h, as part of the standard methodology for this test, after which they were cooled for 24 h in an airtight container. The specimens were then immersed in water for at least 2 days, then removed from the water tank and wiped using a cloth. Weight measurements were taken every 24 h until there was no change in the recorded measurements;

- Crack Width and Depth Tests

The dimensions of cracks (i.e., crack width and crack depth) were measured after performing the destructive tests on cubes and beams (i.e., compressive strength tests and flexural strength tests), respectively. For each specimen, several points were marked at crack openings to measure the crack width at the same points before and after the self-healing of the specimens. A special type of high-resolution camera was used to take the photos, as shown in Figure 3e, which were then analyzed using the software “Portable Capture Plus (v3.1)” to precisely measure the width of the cracks. An ultrasonic test was employed solely on beam specimens to evaluate the crack depths at comparable spots (Figure 3f);

- Microstructure Test

After carrying out the destructive and non-destructive testing on all specimens, tiny samples smaller than 5 mm were retrieved from each sample. A scanning electron microscope (SEM) was then used to investigate the samples to examine the microstructure’s topography. To study and discover more about the self-healing process of the various mixtures, elemental analysis was also carried out.

## 3. Results and Discussion

The findings and discussion resulting from the tests done on the four different concrete mixtures, both containing and excluding crystalline admixtures, are presented in this section and include the concrete’s slump, air content, compressive strength, bending strength, absorption of water, and self-repairing properties. The results offer new insights into the application of crystalline admixtures and coatings in developing long-lasting, environmentally friendly concrete with self-healing capabilities.

The slump test findings on fresh concrete indicate that Mix 2, with a 1% (by weight of cement) crystalline admixture, has the highest slump values, compared to Mix 3, with a 2.5% (by weight of cement) admixture, which exhibits a mild slump. Due to the fact that both mixes are identical in their plastic condition, Mixes 1 and 4 show similar readings and the lowest levels of slump. The initial findings indicate that the crystalline additive enhances the workability of fresh concrete, although more research should be done to establish the ideal dose and establish a link between the dosage amount and concrete workability.

### 3.1. Tests on Fresh-Concrete

#### 3.1.1. Slump Cone Test

A slump test was performed on the fresh concrete, and the findings reveal that Mix 2, with a 1% crystalline admixture has the highest slump values, whereas Mix 3, with a 2.5% crystalline admixture, has a moderate slump (see Table 2). Mixes 1 and 4 showed equal measurements and had the lowest values for slump because both concrete mixtures were comparable in their plastic state. The initial results demonstrate that the crystalline addition improves the workability of fresh concrete, although to determine the appropriate dose and establish a connection between dosage quantity and concrete workability, further research is needed.

#### 3.1.2. Air Content Test

The concrete’s air content is raised by the crystalline admixture in a manner comparable to that shown in the slump test, which followed a similar pattern (see Table 3). The least quantity of air is detected in Concrete Mix 1, whereas the greatest amount is detected in Concrete Mix 2, which contains a 1% crystalline admixture. The results of concrete Mix 3, with a 2.5 percent crystalline admixture, show moderate air content values, suggesting that an ideal admixture dose might be determined.

### 3.2. Tests on Hardened Concrete

#### 3.2.1. Compressive Strength Tests

**Figure 4:**
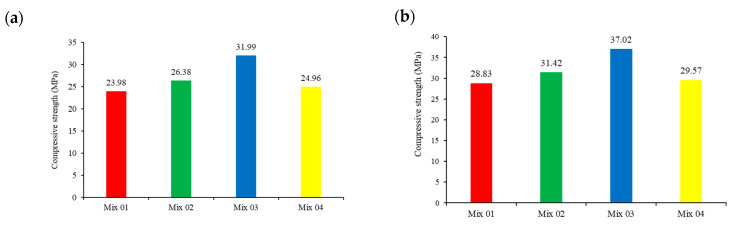
Compressive strength test results: (**a**) non-destructive; and (**b**) destructive.

The compressive strength of each cube specimen is first determined using a non-destructive approach, the rebound hammer test (see Table 4, Figure 4a); a destructive method was subsequently used (see Table 5, Figure 4b). The findings show that destructive testing demonstrated greater strength when compared with non-destructive tests, with an average strength variation of 5 MPa between the two techniques.

The maximum compressive strength was found in Mix 3, followed by Mixes 2, 4, and 1, demonstrating that self-repairing concrete possesses higher compressive strengths than regular concrete. Interestingly, Mix 3 indicates a significant increase in strength (28.38% higher than Mix 1), whereas Mixes 2 and 4 show only small increases of 8.98% and 2.56%, respectively.

The observed enhancement in compressive strength can be attributed to the introduction of a crystalline element into the cement matrix. This introduction prompts the formation of crystals, which subsequently leads to the creation of a denser microstructure and a consequent increase in compressive strength.

#### 3.2.2. Flexural Strength Test

**Figure 5:**
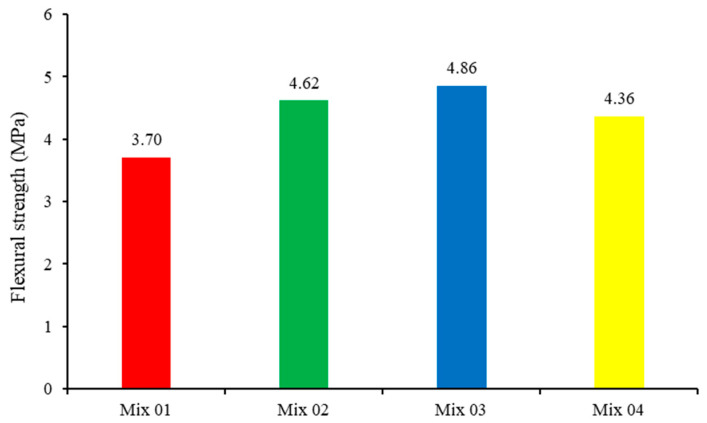
Flexural Strength Test Results.

According to the results of the flexural tests mentioned below in Table 6 and Figure 5, the bending strength of the concrete specimens is improved when more of the crystalline admixture is utilized. Crystalline-coated specimens also demonstrate greater strength than uncoated specimens. Mix 3 outperforms regular concrete in terms of flexural strength, increasing it by 31.17%, while Mix 2 and Mix 4 outperform regular concrete in terms of bending strength, increasing it by 24.73% and 17.72%, respectively.

#### 3.2.3. Water Absorption Test

The outcomes of the water absorption tests are displayed in Table 7, demonstrating that Mix 1 (conventional concrete) has the most water absorption, while Mix 3 (self-repairing concrete with crystalline admixture (2.5%)) has the lowest water absorption. Mix 2 and Mix 4 show intermediate results. The water absorption tests confirm that the crystalline additive has an effect on microstructure compaction in concrete by drastically decreasing water absorption. This suggests that self-repairing concrete tends to be less porous and more resilient than conventional concrete.

#### 3.2.4. Crack Width and Depth Test

An analysis of crack width (see Table 8) and crack depth (see Table 9), shows that Mix 1 does not exhibit any signs of self-healing. Mixes 2, 3 and 4 all demonstrate self-healing properties, with Mix 3 exhibiting the most prominent crack repair characteristics.

The lack of self-healing in Mix 1 may be attributed to two potential factors: delayed crystallization processes or a lower concentration of unhydrated cement in the mixture. In contrast, Mixtures 2, 3, and 4 contain crystalline additives with hydrophilic characteristics. The presence of water causes these crystalline additives to undergo a chemical reaction, which in turn causes the formation of stationary deposits. This effectively seals off any gaps or cracks. The calcium silicate hydrate (CSH) level is significantly increased, and water penetration is effectively hindered by this technique. The incorporation of a crystalline admixture into Mixes 2, 3, and 4 facilitates the formation of insoluble-in-water deposits, thereby increasing their capacity for self-repairing.

#### 3.2.5. Microstructure Test

The specimens were examined using a scanning electron microscope (SEM) at three specific levels of magnification: 200×, 800×, and 1000×. The images obtained at a magnification of 1000× exhibited significantly enhanced clarity and readability and played a large role in achieving our research objectives. The higher magnification level made the enhanced visualization of crystal structures and coatings possible, thereby enabling a comprehensive investigation.

**Figure 6:**
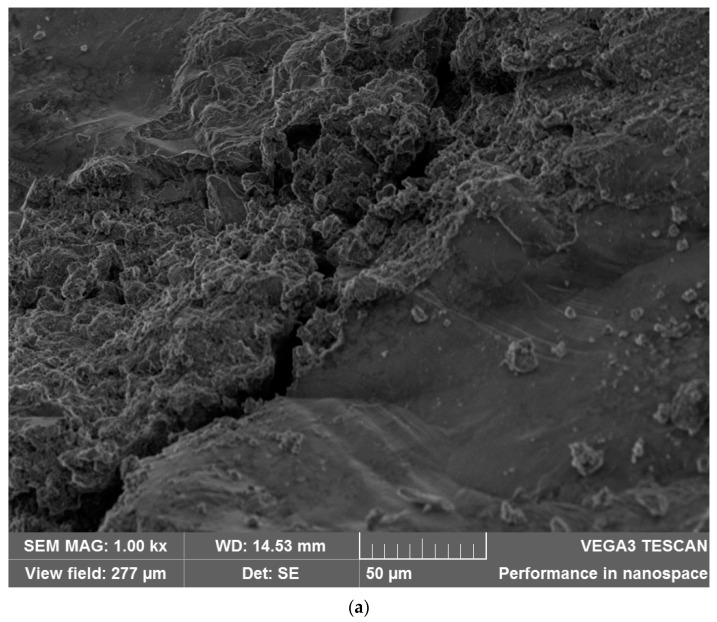

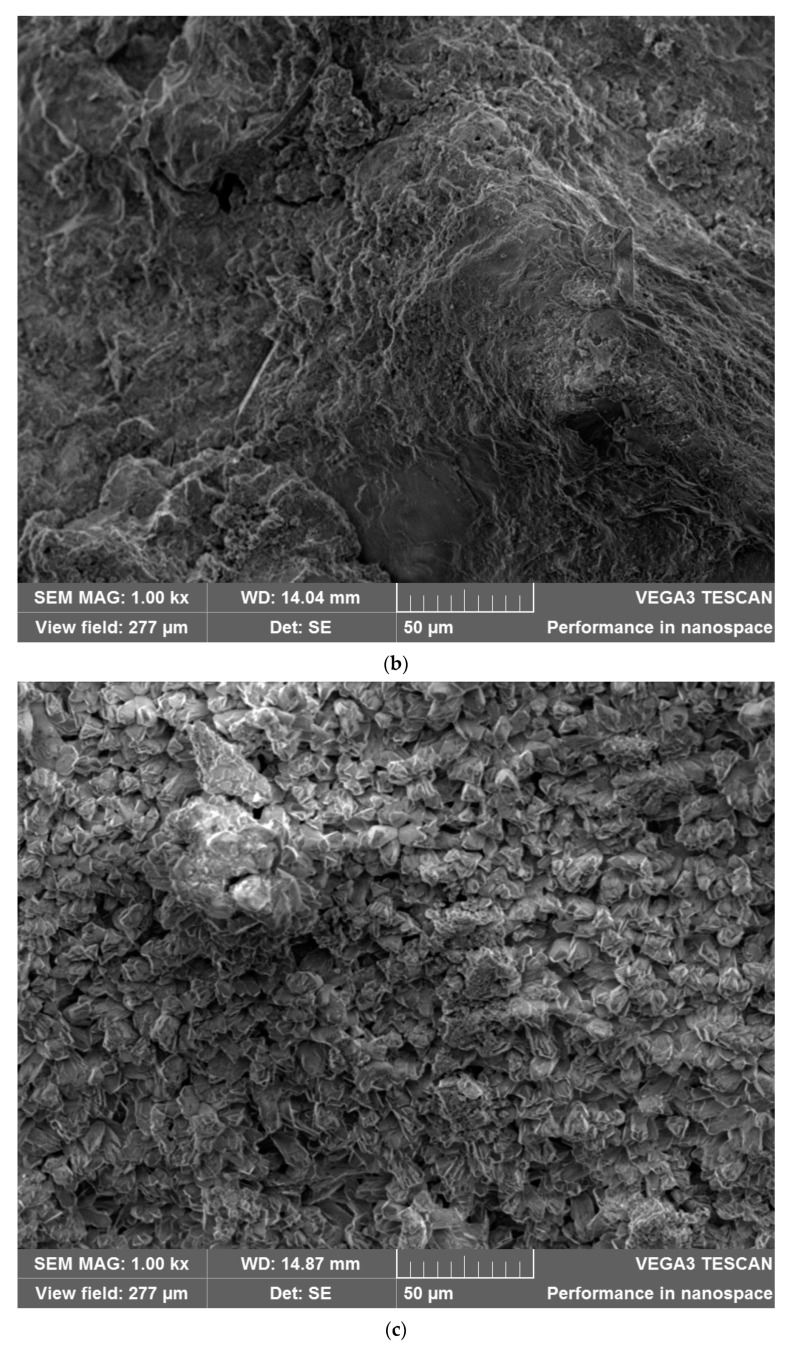

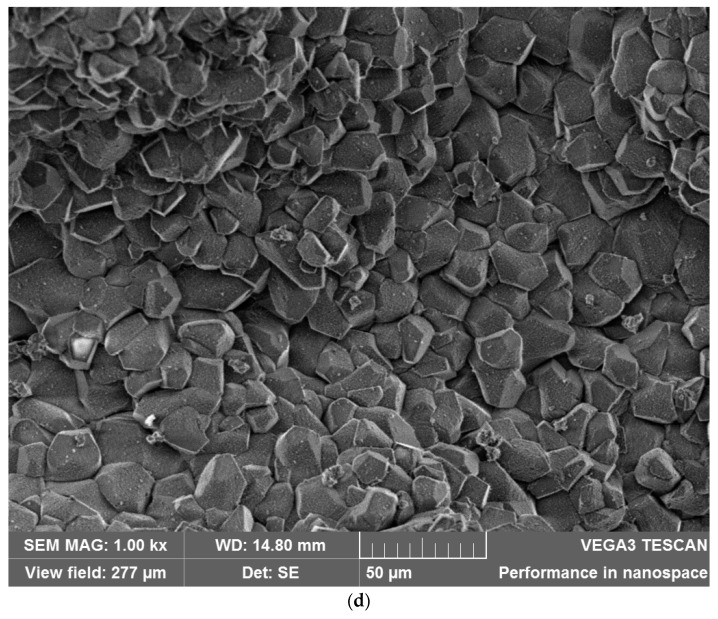
Topographical analysis using SEM: (**a**) Mix 1 (Mag: 1000×); (**b**) Mix 2 (Mag: 1000×); (**c**) Mix 3 (Mag: 1000×); and (**d**) Mix 4 (Mag: 1000×).

The distributions of hydrated materials and hexagonal-shaped calcium compound crystals are more compact and consistent in the self-healing concrete with crystalline admixtures and coatings. A more in-depth examination using SEM showed that the cracks in the self-healing concrete have effectively closed up after the healing process. The crack in Mix 1, however, was only minimally repaired, as seen in the electron microscope photographs (refer to Figure 6). The observed disparity in crack closure behavior indicates that the utilization of CA in this research facilitated the accelerated production of healing chemicals in cracks, thereby enhancing the crack closure process. In addition, larger calcium amounts are observed in the SEM-based elemental analyses of self-repairing concrete compared to ordinary concrete, as indicated in Table 10. This indicates that the crystalline admixtures and coatings improve the concrete’s microstructure, hydration process, and the formation of a cementitious matrix.

**Figure 7:**
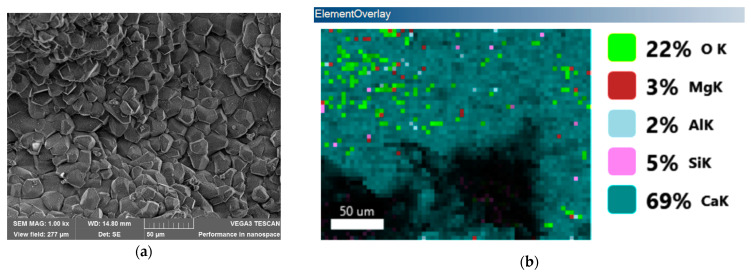

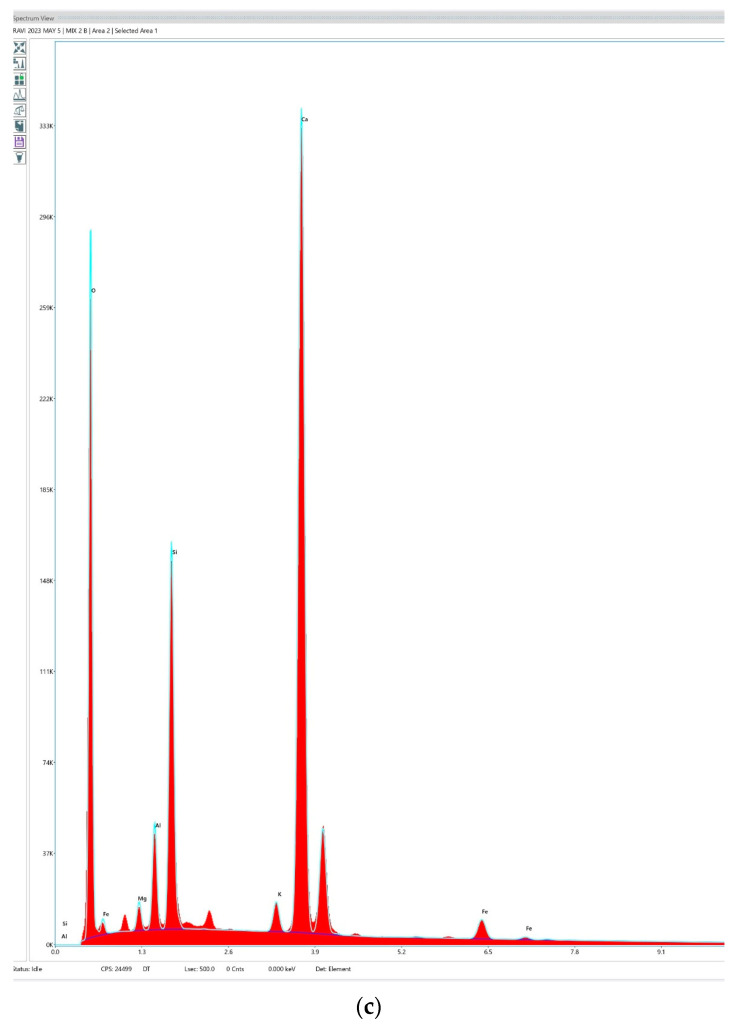
Elemental analysis using SEM: (**a**) topographical analysis; (**b**) elemental analysis; and (**c**) spectrum.

The scanning electron microscope images also confirm the presence of self-repairing crystals (see Figure 7). These crystals are evenly distributed throughout the matrix, confirming their potential role in the bridging and healing of cracks. Ordinary concrete, in contrast, has a less favorable arrangement of hydrated particles and a noticeably looser microstructure, as shown in the electron microscope images.

The SEM investigation supports earlier findings about the improved healing capability of self-repairing concrete employing crystalline admixtures and coatings. A richer microstructure and the presence of a crystalline structure indicates a higher likelihood of self-healed cracks and the emergence of self-healing processes.

## 4. Conclusions and Recommendations

This study provided valuable insights into the efficacy of self-healing concrete by conducting a comparative analysis of four distinct concrete compositions: one conventional mixture and three variations of self-healing concrete.

Distinct patterns in the performance of various combinations were observed through various experiments conducted on both fresh and hardened concrete. The most unfavorable results were observed in traditional concrete, highlighting the insufficiencies of standard combinations in addressing concerns such as crack formation and durability. In contrast, Mix 3, which had a 2.5% concentration of crystalline admixture, demonstrated outstanding performance in all assessed parameters. Mix 4, with the crystalline admix coatings, performed adequately, ranking between the regular mix and the best-performing Mix 3. The discovery implies that the presence of crystalline admixtures and crystalline coatings significantly influences the overall characteristics of self-healing concrete.

In the slump cone and air content testing, concrete Mix 2 performed best, whereas Mix 1 performed the worst. Evidence from this study points to the possibility that crystalline admixtures can enhance the properties of freshly mixed concrete and find practical utility in many contexts. The results of the bending and compressive tests showed that concrete Mix 3 had the highest overall strength values, whereas concrete Mix 1 had the lowest strength. This shows that the strength of concrete is related to the amount of crystalline additive added. The observed enhancement in strength can be attributed to the introduction of a crystalline element into the cement matrix, which prompts the formation of crystals, leading to the creation of a denser microstructure and a consequent increase in strength.

The results of the water absorption test showed that regular concrete absorbed the most water, whereas concrete Mix 3 absorbed the least. It was also discovered that crystalline admixture-added and crystalline admixture-coated concrete have slightly higher weight densities than conventional concrete. The aforementioned findings show that crystalline admixtures can substantially decrease water absorption and increase concrete density, boosting the overall durability and longevity of a structure.

The capabilities and advantages of self-healing concrete over conventional concrete were shown by measurements of crack width, crack depth, and microstructure results for Mixes 2, 3, and 4. As a result, it can be said that self-healing concrete mixes outperform typical concrete mixes in terms of both fresh and hardened properties. This suggests that they may have a significant impact on the building sector in the future.

These results not only indicate positive prospects for future construction, but also showcase the practicality of integrating self-healing concrete with crystalline coatings into existing buildings. By implementing this dual application, we are adopting a practical and adaptable strategy to improving the strength and durability of concrete structures.

In conclusion, there are promising possibilities for the development of self-healing concrete that involve the use of crystalline admixtures, either as internal components of the concrete mix or added as external layers. In addition to paving the way for future research and advancements in self-healing concrete technology, these discoveries significantly contribute to the continuous search for more resilient and environmentally friendly building materials.

To understand more about the use of crystalline admixtures as an additive and coating in self-repairing concrete, further study is required. Comparing the cost and environmental impact of this approach to conventional concrete is necessary to see whether it can be employed on a larger scale. The creation of self-repairing concrete with reinforcement using crystalline admixtures and coatings must also be taken into consideration, since concrete is brittle. It will undoubtedly have a quicker and favorable impact on the construction sector. It may be also possible to speed up the adoption of this breakthrough approach by developing standards and guidelines that regulate how self-repairing concrete should be used in specific conditions.

## Figures and Tables

**Table 1:** Summary of mix designs.

Composition kgm3	Mix 01	Mix 02	Mix 03	Mix 04
Cement	360.0	360.0	360.0	360.0
FA (0–4 mm)	725.0	725.0	725.0	725.0
CA (4–8 mm)	484.0	484.0	484.0	484.0
CA (8–16 mm)	641.0	641.0	641.0	641.0
Crystalline Admixture	No	3.6	9.0	No
Crystalline Coating	No	No	No	Yes
Water	176.4	176.4	176.4	176.4

**Table 2:** Slump cone test results.

Mix	Slump Type	Slump 01 (cm)	Slump 02 (cm)	Slump 03 (cm)	Average Slump (cm)
Mix 01	True	13.7	14.1	15.2	14.3
Mix 02	True	17.2	16.2	16.8	16.7
Mix 03	True	15.6	16.1	14.9	15.5
Mix 04	True	14.8	14.3	13.9	14.3

**Table 3:** Air content test results.

Mix	Air Content 01 (%)	Air Content 02 (%)	Air Content 03 (%)	Average Air Content (%)
Mix 01	1.0	1.3	0.7	1.0
Mix 02	1.8	1.9	1.7	1.8
Mix 03	1.4	1.5	1.6	1.5
Mix 04	0.8	1.0	1.2	1.0

**Table 4:** Non-destructive compressive strength test results.

Mix	Compressive Strength 1 (MPa)	Compressive Strength 2 (MPa)	Compressive Strength 3 (MPa)	Mean Compressive Strength (MPa)
Mix 01	21.15	26.70	24.10	23.98
Mix 02	24.10	24.90	30.15	26.38
Mix 03	33.47	31.85	30.66	31.99
Mix 04	29.10	23.60	22.17	24.96

**Table 5:** Destructive compressive strength test results.

Mix	C. Force P1 (kN)	C. Force P2 (kN)	C. Force P3 (kN)	Mean C. Force (kN)	X-Sectional Area (mm^2^)	Mean C. Strength (MPa)	Percentage Increase in Comparision to Mix 01 (%)
Mix 01	564.75	715.50	666.10	648.78	22,500.00	28.83	-
Mix 02	643.05	676.50	801.50	707.02	22,500.00	31.42	8.98%
Mix 03	870.08	827.00	801.70	832.93	22,500.00	37.02	28.38%
Mix 04	760.90	640.40	594.80	665.37	22,500.00	29.57	2.56%

**Table 6:** Flexural strength test results.

Mix	Flexural Force P1 (kN)	Flexural Force P2 (kN)	Mean Flexural Force (kN)	Mean Flexural Strength (MPa)	Percentage Increase in Comparision to Mix 01 (%)
Mix 01	26.66	28.86	27.76	3.70	-
Mix 02	34.41	34.85	34.63	4.62	24.73%
Mix 03	36.54	36.29	36.42	4.86	31.17%
Mix 04	32.45	32.92	32.68	4.36	17.72%

**Table 7:** Water absorption test results.

Mix	ODM 1 (kg)	ODM 2 (kg)	ODM 3 (kg)	Average ODM (kg)	SSDM 1 (kg)	SSDM 2 (kg)	SSDM 3 (kg)	Average SSDM (kg)	Water Absorption (%)
Mix 01	7.32	7.32	7.34	7.33	7.70	7.68	7.70	7.69	5.00%
Mix 02	7.92	7.76	7.84	7.84	8.10	7.96	8.02	8.03	2.38%
Mix 03	8.16	8.00	8.16	8.11	8.32	8.16	8.32	8.27	1.97%
Mix 04	7.70	7.74	7.66	7.70	7.96	7.94	7.88	7.93	2.94%

**Table 8:** Crack width test results.

Mix	Average Initial Crack Width (mm)	Average Final Crack Width (mm)	Average Crack Healing (mm)	Average Crack Healing (%)
Mix 01	0.952	0.952	0.000	0.00
Mix 02	0.703	0.657	0.047	6.62
Mix 03	0.813	0.728	0.085	10.45
Mix 04	0.387	0.362	0.025	6.55

**Table 9:** Crack depth test results.

Mix	Average Final Crack Depth (mm)	Average Final Crack Depth (mm)	Average Crack Healing (mm)	Average Crack Healing (%)
Mix 01	134	134	0	0.000
Mix 02	130	127	3	2.31
Mix 03	123	116	7	5.69
Mix 04	126	123	3	2.38

**Table 10:** Elemental analysis test results.

Element	Mix 01	Mix 02	Mix 03	Mix 04
O	60%	61.0%	52.3%	64.0%
Na	0.4%	0.0%	0.0%	0.0%
Mg	0.5%	0.0%	0.6%	1.5%
Al	1.8%	0.0%	1.0%	0.5%
Si	10.5%	7.2%	2.7%	0.4%
K	0.4%	0.8%	0.0%	0.0%
Ca	24.7%	31.0%	39.6%	33.6%
Fe	1.1%	0.0%	3.8%	0.0%

## Data Availability

The datasets generated during and/or analyzed during the current study are available from the corresponding author upon reasonable request.
